# Translational Evaluation of a Disodium Adenosine Monophosphate (AMP2Na)-Based Topical Formulation for Physiology-Aligned Skin Rejuvenation: Integrated In Vitro, Ex Vivo, and Clinical Evidence

**DOI:** 10.3390/ijms27114840

**Published:** 2026-05-27

**Authors:** Ngoc Ha Nguyen, Young In Lee, Yoo Jin Kim, Hwiyeong Lee, Jihee Kim, Ju Hee Lee

**Affiliations:** 1 Department of Dermatology & Cutaneous Biology Research Institute, Yonsei University College of Medicine, Seoul 03722, Republic of Korea; nguyenngocha7996@gmail.com (N.H.N.); ylee1124@yuhs.ac (Y.I.L.); 2 Department of Dermatology, University of Medicine and Pharmacy at Ho Chi Minh City, Ho Chi Minh City 700000, Vietnam; 3 Scar Laser and Plastic Surgery Center, Yonsei Cancer Hospital, Seoul 03722, Republic of Korea; 4 Global Medical Research Center, Seoul 06526, Republic of Korea; kyj@gmrc.co.kr (Y.J.K.); hy1101@gmrc.co.kr (H.L.); 5 Department of Dermatology, Yongin Severance Hospital, Yonsei University College of Medicine, Yongin-si 16995, Republic of Korea; mygirljihee@yuhs.ac

**Keywords:** skin aging, adenosine monophosphate, rejuvenation, hyperpigmentation, cellular senescence, wrinkling, antioxidants, extracellular matrix, skin barrier, epidermal turnover

## Abstract

Skin aging stems from intrinsic decline and external stressors that induce oxidative stress and mitochondrial damage, ultimately lowering cellular energy production and slowing epidermal turnover to cause wrinkles, dryness, and pigment imbalances. While disodium adenosine monophosphate (AMP2Na) is hypothesized to enhance cellular adenosine triphosphate production and restore epidermal metabolism, its broader anti-aging effects have remained underexplored. To address this, a multi-tiered study integrating in vitro, ex vivo, and clinical investigations was conducted. Specifically, a 12-week exploratory clinical trial involving female participants with facial hyperpigmentation (n = 23), alongside a short-term forearm study (n = 22), suggested that the AMP2Na-containing product could reduce wrinkles and hyperpigmentation while safely improving hydration, barrier function, skin lifting, and epidermal turnover with high participant satisfaction. Mechanistically, in vitro assays on human dermal fibroblasts showed that the formulation restored antioxidant enzyme activity and mitigated senescence. Ex vivo UVB-irradiated skin explant models corroborated these findings by revealing reduced melanin levels, preserved collagen and elastin networks, and an upregulation of key structural and barrier-related proteins. Ultimately, by potentially supporting epidermal turnover and restoring barrier function through this biomimetic mechanism, the AMP2Na-containing product might offer a promising option for alleviating wrinkles, dryness, and hyperpigmentation. Future randomized, vehicle-controlled clinical trials and comprehensive laboratory studies are warranted to validate its true potential in skin rejuvenation.

## 1. Introduction

Skin aging arises from both intrinsic biological processes and extrinsic environmental factors, particularly chronic ultraviolet (UV) exposure. Intrinsic aging, most evident in sun-protected areas, is characterized by diminished epidermal regeneration driven by reduced keratinocyte stem cell activity and depletion of adenosine triphosphate (ATP) reservoir due to mitochondrial damage, leading to progressive skin thinning [1,2,3]. Both aforementioned pathways converge on excessive generation of reactive oxygen species (ROS), which induce oxidative stress, enzymatic degradation of extracellular matrix (ECM) components, and dysregulated melanogenesis—culminating in wrinkles, laxity, and hyperpigmentation, the latter being especially persistent and challenging to treat [1,4].

Conventional whitening approaches have focused on inhibiting melanin synthesis, particularly through tyrosinase suppression [5,6,7]. While effective at preventing new pigment formation, these methods show limited capacity in increasing skin turnover to remove pre-existing melanin, yield slow visible improvement, and may cause irritation or interfere with the melanin’s natural photoprotective functions [6,7,8,9]. Hence, a physiology-aligned depigmentation strategy aiming to enhance epidermal turnover has become highly appealing to dermatologists.

In this context, adenosine monophosphate (AMP) is a fundamental nucleotide required for the synthesis of DNA, RNA, and ATP, while cyclic AMP (cAMP) serves as a major second messenger that regulates diverse cellular processes [10,11,12]. Naturally present in plant seeds, bulbs, bamboo joints, and breast milk [13,14,15], AMP plays a central role in cellular energy metabolism, driving a stratum corneum renewal cycle of around 20–30 days in young adults [16]. With aging, mitochondrial dysfunction in skin cells leads to diminished ATP production, resulting in a slower epidermal turnover rate of 40–60 days in the elderly and the subsequent accumulation of dead keratinocytes and melanin [3,16,17].

Interestingly, disodium adenosine monophosphate (AMP2Na), a more water-soluble form of AMP, is hypothesized to support DNA synthesis and promote keratinocyte proliferation and turnover by delivering exogenous AMP into the skin to increase intracellular ATP and cAMP levels [18,19,20]. Subsequently, this facilitates the removal of accumulated melanin while preserving the normal melanogenesis, producing a skin-friendly depigmenting effect [18,19,21,22,23]. Although its whitening activity has been documented, the broader benefits of AMP2Na remain insufficiently investigated.

Therefore, we conducted an integrated study to investigate the rejuvenating potential of an AMP2Na-containing product. Specifically, human dermal fibroblasts (HDFs) were utilized in vitro to quantify antioxidant enzyme activities and cellular senescence. Ex vivo UVB-irradiated human skin explants were analyzed to evaluate ECM repair, melanin reduction, and the expression of structural and hydration-related proteins. Translationally, two clinical trials involving females were conducted to assess wrinkle reduction, skin tone improvement, hydration, barrier recovery, soothing effects, and epidermal turnover following the application of an AMP2Na-based emulsion.

## 2. Results

### 2.1. In Vitro Study

**Figure 1:**
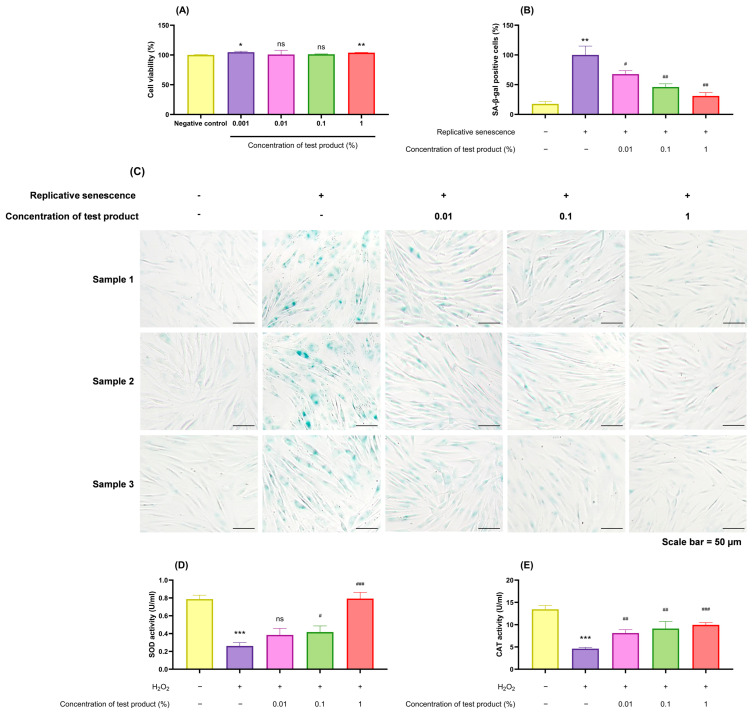
In vitro efficacy of the test product on HDFs. (**A**) Cell viability of HDFs following 24 h exposure to increasing concentrations of the test product. (**B**) Percentage of SA-β-gal-positive cells in different groups of HDFs. (**C**) Representative images of SA-β-gal staining in different groups of HDFs. Activity levels of the antioxidant enzymes SOD (**D**) and CAT (**E**) in different groups of HDFs. Data are presented as mean ± standard deviation. * compared with the negative control group; * *p* < 0.05, ** *p* < 0.01, *** *p* < 0.005. # compared with the replicative senescent/H_2_O_2_-exposed control group; # *p* < 0.05, ## *p* < 0.01, ### *p* < 0.005; ns, not significant. SA-β-gal, Senescence-Associated β-galactosidase; SOD, Superoxide Dismutase; CAT, Catalase.

The cell viability assay showed that the test product induced HDF proliferation and demonstrated a good cellular safety profile (Figure 1A). Based on these results, the three highest non-cytotoxic concentrations (0.01, 0.1, and 1%) were selected for further testing.

After the induction of replicative senescence via over 30 passages, the percentage of senescence-associated β-galactosidase (SA-β-gal)-positive fibroblasts was significantly increased. However, treatment with the test product considerably diminished this effect, with better reductions observed in higher concentrations (Figure 1B). Representative images of SA-β-gal staining further corroborated these findings (Figure 1C)

Hydrogen peroxide (H_2_O_2_)-induced stress markedly reduced the activity of the antioxidant enzymes superoxide dismutase (SOD) and catalase (CAT), whereas treatment with the test product restored their activity in a dose-dependent manner (Figure 1D,E).

### 2.2. Ex Vivo Human Skin Explant UVB-Induced Aging Model

#### 2.2.1. Histological Assessment of Skin Integrity and Dermal Matrix Remodeling

**Figure 2:**
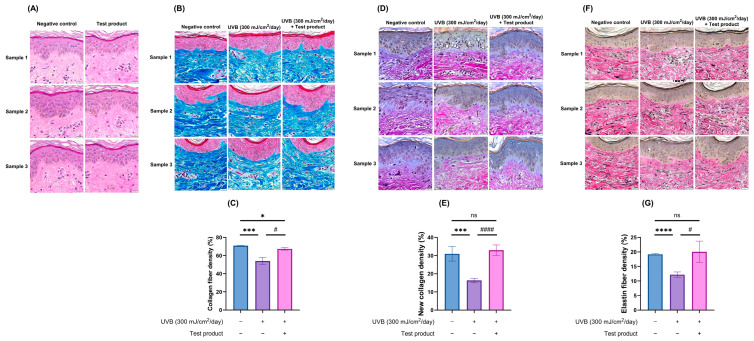
Effects of the test product on ECM components in UVB-irradiated ex vivo human skin explants. (**A**) H&E staining of skin explants showing normal tissue morphology in both the negative control and test product-treated groups. (**B**) Representative images of Masson’s Trichrome staining, where total collagen fibers are stained blue. (**C**) Quantitative analysis of total collagen fiber density. (**D**) Representative images of HV staining, identifying newly synthesized collagen (blue) versus mature collagen (red). (**E**) Quantitative analysis of the percentage of new collagen. (**F**) Representative images of VVG staining, where elastin fibers are stained black. (**G**) Quantitative analysis of elastin fiber density. All scale bars = 20 µm. Data are presented as mean ± standard deviation. * compared with the negative control group; * *p* < 0.05, *** *p* < 0.005, **** *p* < 0.001; ns, not significant. # compared with the UVB-irradiated group; # *p* < 0.05, #### *p* < 0.001. UVB, ultraviolet B.

Hematoxylin and eosin (H&E) staining demonstrated the safety of the test product when the integrity of both the epidermis and dermis was preserved, with no signs of tissue disruption and inflammation similar to the negative control group (Figure 2A).

Masson’s Trichrome (MT) staining revealed that UVB exposure induced significant degradation and disorganization of the dermal collagen network, resulting in reduced collagen fiber density. In contrast, treatment with the test product preserved a dense and organized collagen matrix, closely resembling that of the negative control (Figure 2B,C).

Herovici (HV) staining demonstrated that UVB exposure markedly suppressed neocollagenesis, whereas treatment with the test product reversed this inhibition and significantly promoted new collagen production to levels comparable with the negative control (Figure 2D,E).

Verhoeff–Van Gieson (VVG) staining showed that UVB exposure markedly disrupted the elastin network, leading to reduced fiber density and clumped, fragmented structures characteristic of solar elastosis. In contrast, treatment with the test product mitigated this damage and preserved a more organized and dense elastin architecture (Figure 2F,G).

#### 2.2.2. Melanin Reduction

**Figure 3:**
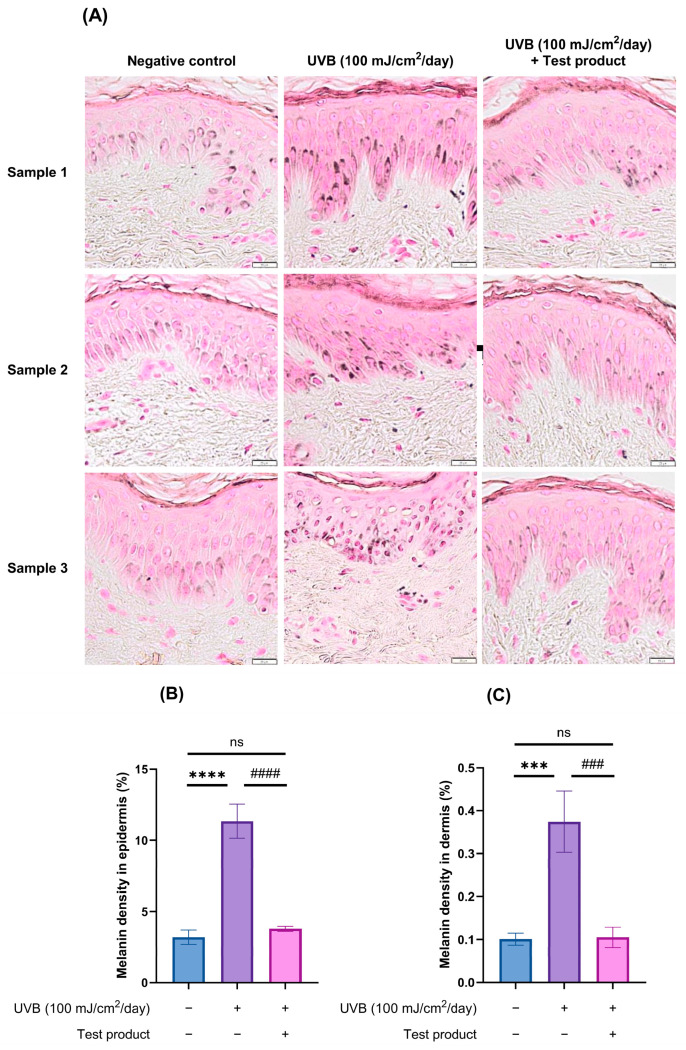
Effect of the test product on melanin deposition in UVB-irradiated ex vivo human skin explants. (**A**) Representative images of Fontana–Masson staining, which visualizes melanin as black deposits within the skin tissue. Quantitative analysis of melanin density in the epidermis (**B**) and the dermis (**C**). All scale bars = 20 µm. Data are presented as mean ± standard deviation. * compared with the negative control group; *** *p* < 0.005, **** *p* < 0.001, ns, not significant. # compared with the UVB-irradiated group; ### *p* < 0.005, #### *p* < 0.001. UVB, ultraviolet B.

Representative Fontana–Masson (FM) staining images showed that UVB exposure increased melanin deposition in both the epidermis and dermis, whereas treatment with the test product significantly reduced melanin levels in both compartments to values comparable to those of the control (Figure 3A). Quantitative analysis further supported these findings (Figure 3B,C).

#### 2.2.3. Effects on Markers of Cell Proliferation and Skin Barrier Components

**Figure 4:**
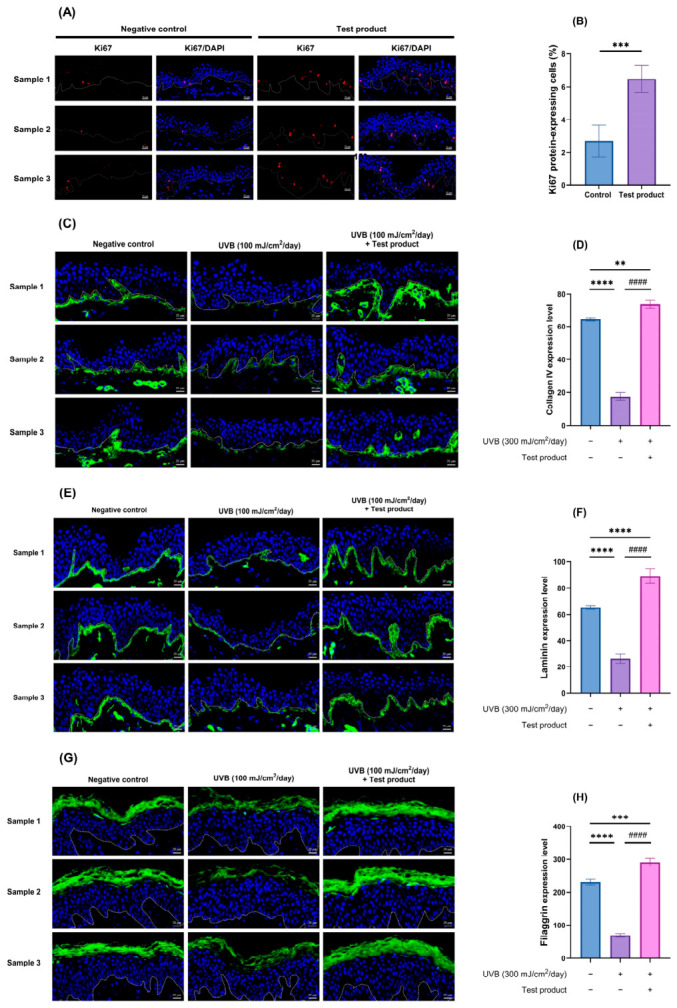
Effects of the test product on cell proliferation and skin barrier protein expression in UVB-irradiated ex vivo human skin explants. Protein localization and expression were visualized via immunofluorescence (IF) staining, with nuclei counterstained using DAPI (blue). (**A**) Representative IF images of Ki67 expression (red) within the basal layer of the epidermis, serving as a marker for mitotic activity. (**B**) Quantitative assessment of Ki67-positive cells, expressed as a percentage of total basal keratinocytes. (**C**) Representative IF imaging of Collagen IV (green) localized along the basement membrane zone. (**D**) Densitometric quantification of Collagen IV intensity, reflecting the structural integrity of the dermal–epidermal junction (DEJ). (**E**) Visualization of Laminin expression (green) at the DEJ interface. (**F**) Quantitative analysis of Laminin fluorescence intensity at the DEJ. (**G**) Representative IF images of Filaggrin (green) distribution within the stratum corneum, indicating epidermal differentiation and barrier function. (**H**) Quantitative analysis of Filaggrin expression levels. All scale bars = 20 µm. Data are presented as mean ± standard deviation. * compared with the negative control group; ** *p* < 0.01, *** *p* < 0.005, **** *p* < 0.001. # compared with the UVB-irradiated group; #### *p* < 0.001. UVB, ultraviolet B; DAPI, 4′,6-diamidino-2-phenylindole.

Immunofluorescence (IF) showed that the test product significantly promoted epidermal cell proliferation, as evidenced by a greater number of Ki67-positive nuclei in the basal layer of the epidermis compared with the control [24] (Figure 4A,B). The test product protected the dermal–epidermal junction (DEJ) by preserving key basement membrane proteins. Specifically, UVB exposure markedly degraded collagen IV and laminin, both of which are essential for anchoring and structural integrity [25], whereas the treatment group maintained strong expression of these proteins at levels comparable to those of the negative control, thereby reinforcing DEJ integrity (Figure 4C–F). Moreover, the product partially enhanced the skin’s moisture barrier by preserving filaggrin expression [26]. While UVB markedly reduced filaggrin levels, treatment prevented this loss and thereby supported skin hydration (Figure 4G,H).

**Figure 5:**
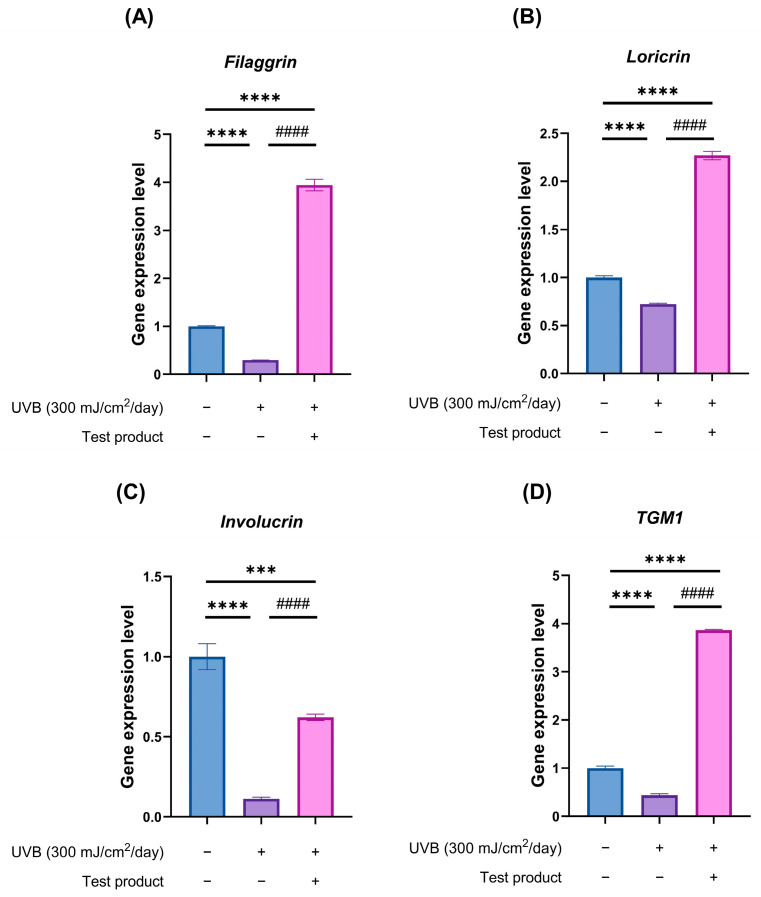
Effects of the test product on skin barrier gene expression in UVB-irradiated ex vivo skin explants. Gene expression levels were determined by quantitative real-time polymerase chain reaction. The graphs show the relative gene expression levels of (**A**) *Filaggrin*, (**B**) *Loricrin*, (**C**) *Involucrin*, and (**D**) *TGM1*. Data are presented as mean ± standard deviation. * compared with the negative control group; *** *p* < 0.005, **** *p* < 0.001. # compared with the UVB-irradiated group; #### *p* < 0.001. UVB, ultraviolet B; *TGM1*, *Transglutaminase 1*.

Quantitative real-time polymerase chain reaction (qRT-PCR) analysis corroborated the IF findings, demonstrating that UVB irradiation markedly suppressed the mRNA expression of *filaggrin*, *loricrin*, *involucrin*, and *transglutaminase 1 (TGM1)*. These biomarkers are pivotal for maintaining cornified envelope integrity and epidermal moisture retention [26]. Notably, treatment with the test product effectively restored the expression of these skin barrier-related genes, suggesting a potent capacity to mitigate UVB-induced damage and promote the recovery of epidermal barrier function (Figure 5A–D).

**Figure 6:**
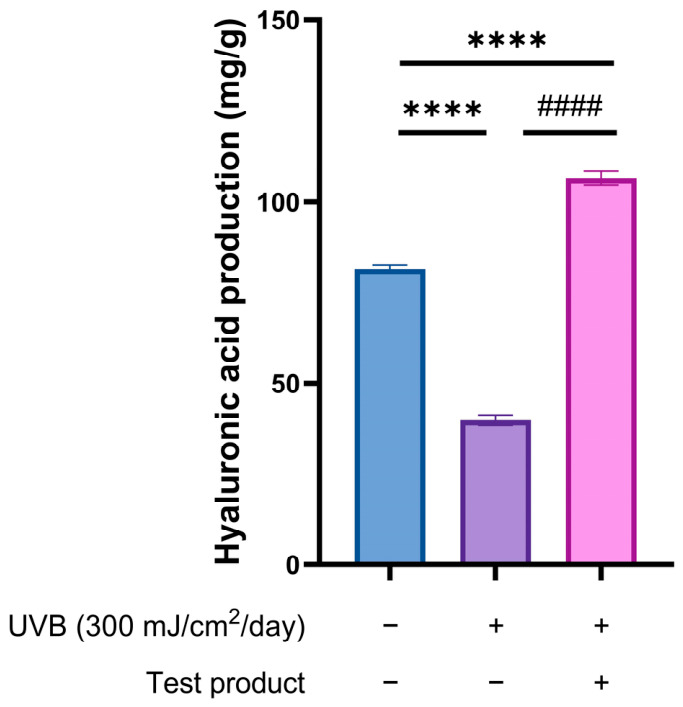
Effects of the test product on Hyaluronic Acid synthesis using Enzyme-Linked Immunosorbent Assay. * compared with the negative control group; **** *p* < 0.001. # compared with the UVB-irradiated group; #### *p* < 0.001. UVB, ultraviolet B.

Enzyme-Linked Immunosorbent Assay (ELISA) further supported the efficacy of the test product in reconstituting hyaluronic acid (HA) levels. As a primary glycosaminoglycan responsible for skin hydration, HA was significantly depleted following UVB irradiation; however, treatment with the test product effectively reversed this loss, suggesting a hydro-protective effect (Figure 6).

### 2.3. Clinical Trial 1: Evaluation of Anti-Aging and Skin Tone Efficacy

Given the rejuvenative, antioxidant, depigmenting properties, and barrier-supportive effects of the test product observed in cellular and human skin tissue models, we next performed two exploratory clinical studies to examine its translational potential in humans with different signs of photoaging and its soothing efficacy.

This exploratory trial recruited 23 participants, with a mean age of 51.609 ± 5.391 years old. All participants had a 100% compliance rate and completed the study.

#### 2.3.1. Wrinkle Reduction

**Figure 7:**
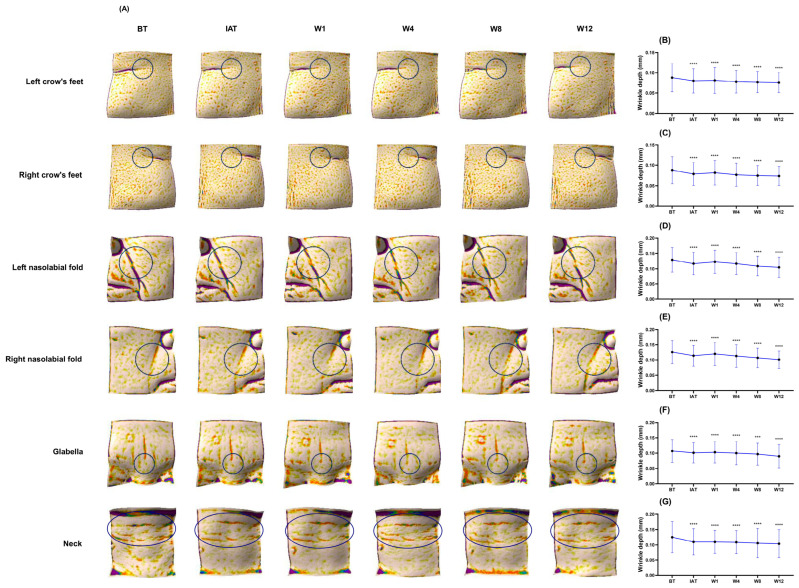
Clinical efficacy of the test product on wrinkle reduction in upper and middle face and neck regions. (**A**) Representative 3D reconstructed images of the skin surface showing wrinkle improvement in the upper and middle face and neck regions over the 12-week study period. The circles mark the area of improvements, with less pronounced shadowing in skin furrows indicating reduced wrinkle depth. Quantitative analysis of the change in wrinkle depth corresponding to representative changes in panel (**A**) over 12 weeks for (**B**) Left crow’s feet, (**C**) Right crow’s feet, (**D**) Left nasolabial fold, (**E**) Right nasolabial fold, (**F**) Glabella, and (**G**) Neck. Data are presented as mean ± standard deviation. * compared with the BT group; *** *p* < 0.005, **** *p* < 0.001. BT, before treatment; IAT, immediately after treatment; W, week.

Application of the test product resulted in a visible and progressive reduction in wrinkle depth (mm) across all measured facial and neck areas over the 12-week study period (Figure 7A).

Wrinkle depth in both crow’s feet areas decreased significantly after the first application and continued to decline through week (W) 12 (left, before treatment (BT), 0.088 ± 0.035; immediately after treatment (IAT), 0.080 ± 0.030; W1, 0.081 ± 0.032; W4, 0.078 ± 0.028; W8, 0.077 ± 0.026; W12, 0.076 ± 0.025; Figure 7B); (right, BT, 0.088 ± 0.033; IAT, 0.079 ± 0.028; W1, 0.082 ± 0.030; W4, 0.077 ± 0.028; W8, 0.075 ± 0.024; W12, 0.074 ± 0.023; Figure 7C).

The depth of both nasolabial folds decreased significantly immediately after treatment and remained reduced at 1, 4, 8, and 12 weeks versus baseline (left, BT, 0.129 ± 0.040; IAT, 0.117 ± 0.037; W1, 0.123 ± 0.038; W4, 0.117 ± 0.036; W8, 0.109 ± 0.032; W12, 0.105 ± 0.033; Figure 7D); (right, BT, 0.126 ± 0.038; IAT, 0.114 ± 0.034; W1, 0.120 ± 0.038; W4, 0.113 ± 0.037; W8, 0.107 ± 0.032; W12, 0.101 ± 0.029; Figure 7E).

Similar trends were observed for glabella wrinkles (BT, 0.107 ± 0.037; IAT, 0.101 ± 0.033; W1, 0.103 ± 0.035; W4, 0.100 ± 0.038; W8, 0.097 ± 0.036; W12, 0.090 ± 0.038; Figure 7F), and neck wrinkles (BT, 0.125 ± 0.051; IAT, 0.110 ± 0.043; W1, 0.110 ± 0.038; W4 weeks, 0.109 ± 0.038; W8, 0.106 ± 0.048; W12, 0.104 ± 0.046; Figure 7G).

#### 2.3.2. Melanin Reduction and Skin Brightness Enhancement

**Figure 8:**
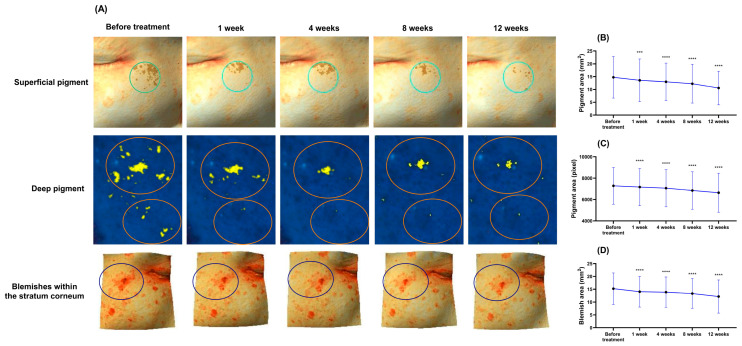
Clinical efficacy of the test product on melanin deposition. (**A**) Representative images showing the reduction in superficial pigment, deep pigment, and blemishes within the stratum corneum over 12 weeks. Top row: the circled areas demonstrate a reduction in the size, intensity, and density of pigmented lesions, corresponding to the quantitative decrease in superficial pigment area in panel (**B**). Middle row: the circled regions show a progressive reduction in signal intensity and number of fluorescent pigment clusters, corresponding to the quantitative decrease in deep pigment in panel (**C**). Bottom row: the circled regions show reduced density and distribution of pigmented corneocyte clusters, consistent with the quantitative reduction in blemish area in panel (**D**). Data are presented as mean ± standard deviation. * compared with the “Before treatment” group; *** *p* < 0.005, **** *p* < 0.001.

The test product demonstrated comprehensive efficacy in reducing skin hyperpigmentation over 12 weeks, with effects observed in superficial and deeper pigment compartments, including blemishes within the stratum corneum (Figure 8A).

Visible superficial pigment spots (mm^2^) showed a significant immediate reduction after application, followed by a continuous decline through W12 versus baseline (BT, 14.739 ± 8.086; W1, 13.565 ± 8.311; W4, 13.000 ± 7.330; W8, 12.261 ± 7.581; W12, 10.565 ± 6.563; Figure 8B).

Consistent reductions were also observed in deep pigment (pixels) (BT, 7280.739 ± 1719.030; W1, 7168.261 ± 1733.740; W4, 7062.522 ± 1742.331; W8, 6842.174 ± 1758.884; W12, 6637.391 ± 1831.617; Figure 8C), and stratum corneum blemishes (mm^2^) (BT, 15.217 ± 6.149; W1, 14.043 ± 5.988; W4, 13.870 ± 5.888; W8, 13.348 ± 5.781; W12, 12.174 ± 6.464; Figure 8D).

**Figure 9:**
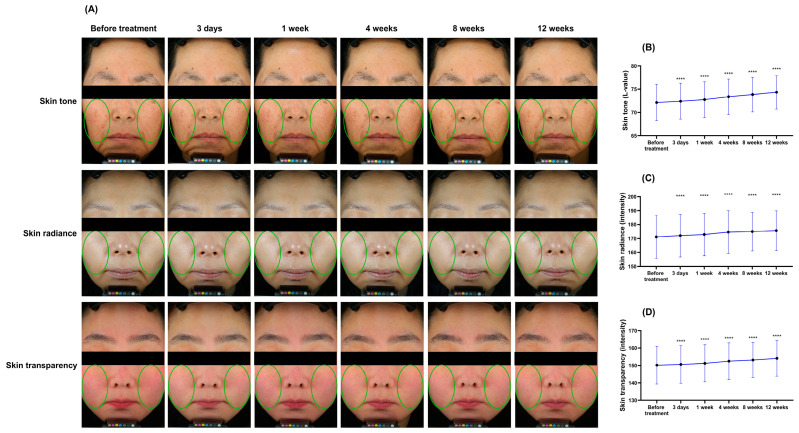
Clinical efficacy of the test product on skin tone, radiance, and transparency. (**A**) Representative clinical photographs illustrating the improvement in skin tone, radiance, and transparency over the 12-week study period. Top row (standard light): the circled areas show a gradual reduction in visible hyperpigmented areas and a more uniform skin color distribution, corresponding to the quantitative increase in skin tone in panel (**B**). Middle row (parallel-polarized light): the circled areas demonstrate a progressive increase in light reflectance and surface brightness, with fewer dull areas, corresponding to the quantitative increase in skin radiance in panel (**C**). Bottom row (cross-polarized light): the circled areas reveal a reduction in blotchy erythema and uneven subsurface coloration, resulting in a more even appearance, which corresponds to the quantitative increase in skin transparency in panel (**D**). Data are presented as mean ± standard deviation. * compared with the “Before treatment” group; **** *p* < 0.001.

In line with pigmentation improvements, the test product enhanced overall skin brightness and luminosity, as shown in clinical photographs (Figure 9A). Specifically, consistent boosts were observed throughout the 12-week period in skin tone (BT, 72.145 ± 3.922; 3 days, 72.419 ± 3.869; W1, 72.772 ± 3.838; W4, 73.375 ± 3.817; W8, 73.841 ± 3.717; W12, 74.329 ± 3.619; Figure 9B), skin radiance (BT, 171.159 ± 15.569; 3 days, 172.044 ± 15.266; W1, 172.864 ± 15.260; W4, 174.543 ± 15.343; W8, 174.948 ± 13.843; W12, 175.589 ± 14.261; Figure 9C), and skin transparency (BT, 150.168 ± 10.783; 3 days, 150.606 ± 10.776; W1, 151.226 ± 10.622; W4, 152.478 ± 10.523; W8, 153.154 ± 10.113; W12, 154.098 ± 10.132; Figure 9D).

#### 2.3.3. Effects on Skin Hydration, Barrier Function, Exfoliation, and Texture

**Figure 10:**
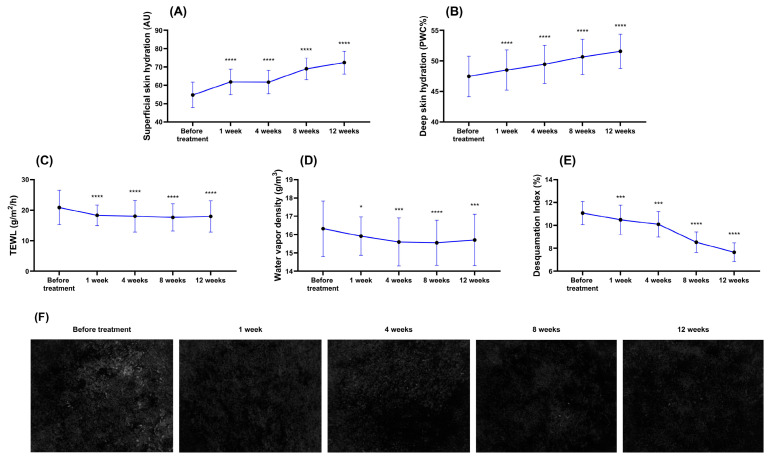
Clinical efficacy of the test product on skin hydration, barrier function, and exfoliation. Quantitative analysis of the change in superficial skin hydration (**A**), deep skin hydration (**B**), TEWL (**C**), and water vapor density (**D**) over 12 weeks. (**E**) Quantitative analysis of the Desquamation Index. (**F**) Representative images of desquamated keratinocytes showing improved skin exfoliation over the 12-week study period. Data are presented as mean ± standard deviation. * compared with the “Before treatment” group; * *p* < 0.05, *** *p* < 0.005, **** *p* < 0.001. TEWL, transepidermal water loss.

The test product significantly improved key aspects of skin health—hydration, barrier integrity, and exfoliation. There were statistically significant increases, sustained through W12, in both superficial skin hydration (AU) (BT, 54.742 ± 6.975; W1, 61.859 ± 7.043; W4, 61.744 ± 6.449; W8, 69.004 ± 5.889; 12 weeks, 72.4 ± 6.253; Figure 10A), and deep skin hydration (%PWC) (BT, 47.471 ± 3.339; W1, 48.503 ± 3.298; W4, 49.438 ± 3.153; W8, 50.667 ± 2.897; W12, 51.586 ± 2.818; Figure 10B).

In parallel, transepidermal water loss (TEWL, g/m^2^h) (BT, 20.883 ± 5.625; W1, 18.284 ± 3.398; W4, 18.007 ± 5.159; W8, 17.610 ± 4.473 g/m^2^h; W12, 17.934 ± 5.109; Figure 10C), water vapor density (g/m^3^) (BT, 16.317 ± 1.510; W1, 15.916 ± 1.041; W4, 15.596 ± 1.308; W8, 15.555 ± 1.229; W12, 15.707 ± 1.404; Figure 10D), and the desquamation index (%) (BT, 11.075 ± 1.017; W1, 10.493 ± 1.284; W4, 10.094 ± 1.112; W8, 8.534 ± 0.895; W12, 7.657 ± 0.818; Figure 10E) all decreased significantly. Representative images show large clumps of corneocytes before treatment being replaced by a finer, more uniform pattern after 12 weeks (Figure 10F).

**Figure 11:**
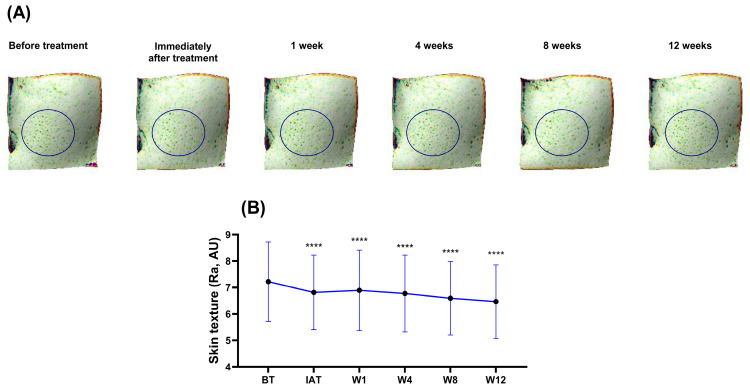
Clinical efficacy of the test product on skin texture. (**A**) Representative images showing the improvement in skin surface texture over the 12-week study period. Over time, the circled areas show progressive smoothing of the skin surface, characterized by reduced surface irregularities, and more uniform distribution of peaks (light areas) and valleys (dark areas). (**B**) Quantitative analysis of the change in skin texture (Ra) corresponding to representative changes in panel (**A**). Data are presented as mean ± standard deviation. * compared with the BT group; **** *p* < 0.001. AU, arbitrary unit. BT, before treatment; IAT, immediately after treatment; W, week.

Aligning with those improvements, the skin texture also demonstrated visible enhancements over 12W (Figure 11A). The Ra index (AU) progressively decreased and remained significantly lower than baseline at all time points (BT, 7.217 ± 1.503; IAT, 6.814 ± 1.409; W1, 6.893 ± 1.520; W4, 6.773 ± 1.450; W8, 6.594 ± 1.388; W12, 6.464 ± 1.393; Figure 11B).

#### 2.3.4. Mid-Face Lifting

**Figure 12:**
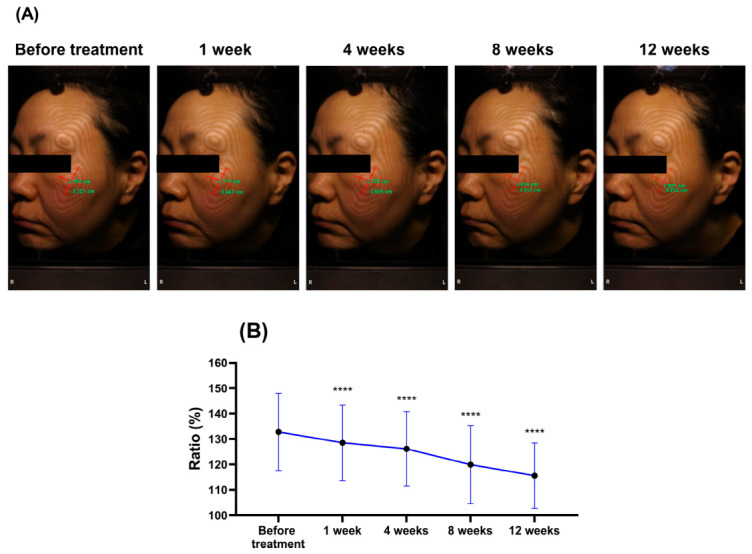
Clinical efficacy of the test product on mid-face lifting. (**A**) Representative Moiré topography images illustrating improvement in mid-face contour over the 12-week study period. The values shown above each image represent the vertical vector, while those below indicate the horizontal vector. (**B**) Quantitative assessment of mid-face lifting, expressed as the ratio of the horizontal vector to the vertical vector derived from panel (**A**). A reduction in this ratio reflects an upward lifting effect in the mid-face region. Data are presented as mean ± standard deviation. **** *p* < 0.001 vs. before treatment.

Aging-induced tissue sagging typically disrupts the youthful oval face shape by increasing the horizontal-to-vertical ratio of the second contour line. Following treatment, this ratio significantly decreased, reflecting a measurable mid-face lifting effect visualized via Moiré topography (Figure 12A). Quantitative analysis (%) confirmed significant, sustained contour improvement from W1 through W12 relative to baseline (BT, 132.762 ± 15.174; W1, 128.527 ± 14.871; W4, 126.118 ± 14.62; W8, 119.938 ± 15.351; W12, 115.565 ± 12.851; Figure 12B).

#### 2.3.5. Subjective Evaluation by Participants

From W1 to W12, subjective questionnaire assessments demonstrated consistently high rates of perceived improvement across multiple parameters among the majority of participants (Table 1). Although a small proportion initially reported no noticeable changes, this proportion progressively declined over the course of the study. No adverse events were reported during the study.

### 2.4. Clinical Trial 2: Evaluation of Soothing Efficacy and Skin Turnover

The second exploratory, prospective, single-center, clinical trial, which aimed to assess the soothing effects and enhancement of skin turnover, enrolled 22 participants (mean age 48.96 ± 10.86 years), all of whom completed it, with compliance rates of 92.86% (left forearm) and 100% (right forearm).

**Figure 13:**
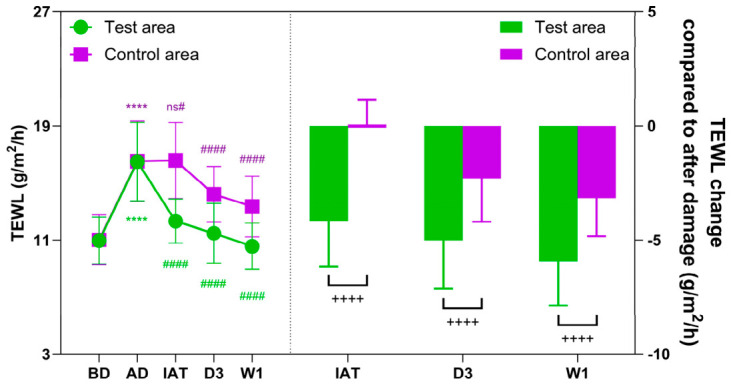
Clinical efficacy of the test product on skin barrier recovery. Quantitative values of TEWL (left graph) and the change in TEWL (right graph) over one week. Data are presented as mean ± standard deviation. * compared with before damage; **** *p* < 0.001. # compared with after damage; #### *p* < 0.001; ns#, not significant. + compare between the test and control areas; ++++ *p* < 0.001. TEWL, transepidermal water loss; BD, before damage; AD, after damage; IAT, immediately after treatment; D, day; W, week.

The test product calmed irritated skin and restored barrier function after being physically compromised. Specifically, TEWL in the test area decreased immediately and progressively over 1W (before damage, 10.974 ± 1.642 g/m^2^/h; after damage, 16.494 ± 2.749 g/m^2^/h; immediately after treatment, 12.334 ± 1.546 g/m^2^/h; 3 days, 11.482 ± 2.091 g/m^2^/h; 1 week, 10.573 ± 1.622 g/m^2^/h), while the control showed no improvement until day 3 (before damage, 11.024 ± 1.764 g/m^2^/h; after damage, 16.517 ± 2.812 g/m^2^/h; immediately after treatment, 16.578 ± 2.658 g/m^2^/h; 3 days, 14.216 ± 1.938 g/m^2^/h; 1 week, 13.357 ± 2.127 g/m^2^/h) (Figure 13. Comparative analysis confirmed that the test area experienced a significantly greater reduction in TEWL than the control area at all time points (Figure 13).

**Figure 14:**
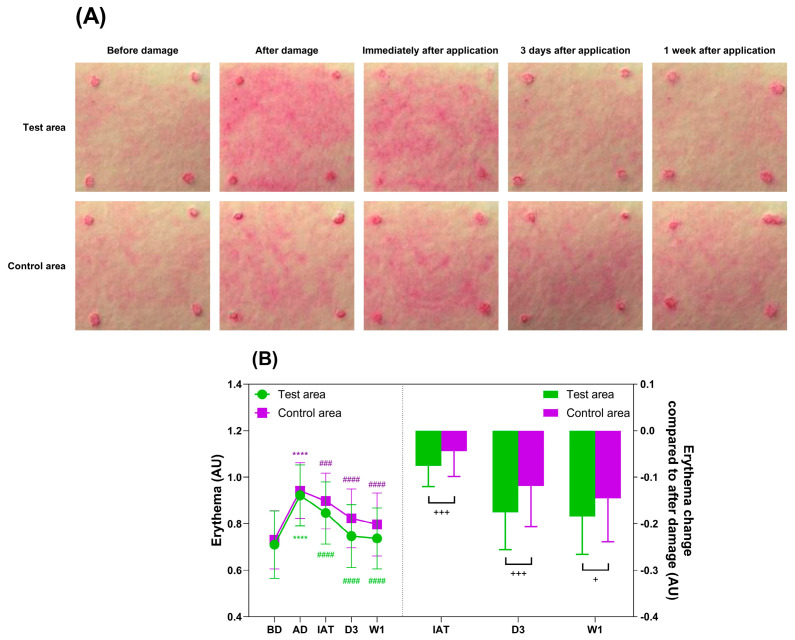
Clinical efficacy of the test product on skin erythema. (**A**) Representative photographs showing greater erythema reduction in the test area compared with the control area. (**B**) Quantitative values of erythema (left graph) and the change in erythema (right graph) over one week. Data are presented as mean ± standard deviation. * compared with before damage; **** *p* < 0.001. # compared with after damage; ### *p* < 0.005, #### *p* < 0.001. + compare between the test and control areas; + *p* < 0.05, +++ *p* < 0.005. BD, before damage; AD, after damage; IAT, immediately after treatment; D, day; W, week.

Erythema (AU) in the treated area was visibly reduced after a single application and sustained for 1 week (test area: before damage, 0.710 ± 0.145; after damage, 0.922 ± 0.131; IAT, 0.846 ± 0.134; 3 days, 0.747 ± 0.135; W1, 0.737 ± 0.131; control area: before damage, 0.731 ± 0.125; after damage, 0.942 ± 0.120; IAT, 0.898 ± 0.120; 3 days, 0.823 ± 0.126; W1, 0.797 ± 0.135; Figure 14A,B), with erythema diminishing faster than in the control at all time points (Figure 14B).

**Figure 15:**
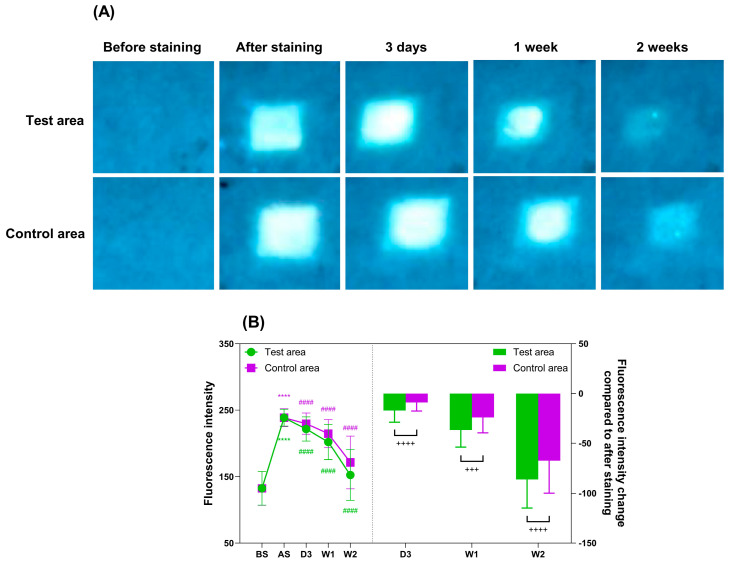
Clinical efficacy of the test product on skin turnover rate. (**A**) Representative images demonstrating greater fading of fluorescent stain in the test area compared with the control over 2 weeks, suggesting a faster turnover rate in the test area. (**B**) Quantitative analysis of skin turnover rate over two weeks, demonstrated as the fluorescence intensity (left graph) and the change in fluorescence intensity (right graph). Data are presented as mean ± standard deviation. * compared with before staining; **** *p* < 0.001. # compared with after staining; #### *p* < 0.001. + compare between the test and control areas; +++ *p* < 0.005, ++++ *p* < 0.001. BS, before staining; AS, after staining; D, day; W, week.

Regarding skin turnover, staining images showed faster dye fading in the test area than the control (Figure 15A). Particularly, the fluorescence decline rate was significantly greater at all follow-up points (test area: before staining, 132.645 ± 25.389; after staining, 238.651 ± 12.782; 3 days, 221.584 ± 18.130; W1, 201.990 ± 26.422; W2, 152.550 ± 38.015; control area, before staining, 132.470 ± 25.493; after staining, 238.654 ± 13.549; 3 days, 229.692 ± 15.920; W1, 214.572 ± 21.236; W2, 171.385 ± 39.588; Figure 15B), indicating accelerated turnover with the test product.

The subjective assessment via questionnaire displayed high rates of perceived relief of skin irritation from the majority of participants (Table 2). Some participants initially reported no difference, but this number progressively decreased over a week. No treatment-related adverse events were observed.

## 3. Discussion

Skin aging is fundamentally driven by decelerated cellular turnover, linked to depleted intracellular energy reserves [3]. As a metabolic mediator, AMP sustains cellular regeneration [10,11,12]. Notably, AMP2Na is a more water-soluble derivative that preserves the biological potency of its parent molecule [18,19,20], consistent with our findings showing its enhancement in the proliferation of HDFs and epidermal cells. Our experiments also showed protective effects of the AMP2Na-containing product against UVB-induced damage, evidenced by reduced melanin deposition and restored barrier integrity. Clinically, these benefits translated into diminished wrinkles, improved facial contours, and sustained hydration. To our knowledge, this represents the first trial to evaluate the multifaceted efficacy of an AMP2Na-containing formulation, validating its potential from bench to clinical application.

Esthetically, reduced melanin levels enhance skin tone, radiance, and transparency [27,28,29]. In our 12-week trial, the formulation significantly diminished superficial and deep pigmented spots, improving all three brightness parameters. This effect is likely driven by accelerated melanin clearance resulting from the enhanced epidermal turnover and increased Ki-67-positive cell proportions observed in our second trial and ex vivo study, respectively. These outcomes may reflect the metabolic potential of AMP2Na and align with a previous 16-week Japanese trial reporting similar improvements in skin turnover and luminosity following twice-daily application of 3% AMP2Na lotion [19].

Beyond delayed turnover, skin senescence is driven by UV-induced ROS production, which promotes fibroblast aging and dermal ECM disorganization. This oxidative stress reduces collagen synthesis and triggers the accumulation of irregular elastin, leading to wrinkles and loss of elasticity [30,31]. Our study demonstrates that the test product bolsters endogenous defenses by restoring SOD and CAT activities and maintaining redox homeostasis. Specifically, SOD dismutates superoxide radicals into hydrogen peroxide, which is subsequently converted into water and oxygen by CAT, potentially helping protect mitochondria and fibroblasts from oxidative senescence [30,32]. Coupled with AMP2Na-induced fibroblast proliferation, these processes likely drive ECM regeneration, as evidenced by restored collagen and elastin architecture ex vivo. These structural improvements could underpin the clinically observed wrinkle reduction and measurable mid-face lifting effects, indicative of enhanced firmness and contour. However, future research into the Nrf2/KEAP1/ARE signaling cascade is warranted to further elucidate the molecular mechanisms behind AMP2Na’s antioxidant enhancement.

In the skin barrier, loricrin and involucrin are key structural proteins of the cornified cell envelope, while filaggrin supplies natural moisturizing factors essential for epidermal hydration [33]. TGM1 further stabilizes the barrier through enzymatic cross-linking [26]. At the DEJ, collagen IV and laminin form the basement membrane that anchors the epidermis and maintains skin stability, whereas HA regulates hydration in both the epidermis and dermis [25,34,35]. During senescence and after UV-induced damage, depletion of these moisturizing components together with DEJ disruption leads to reduced hydration, increased water loss, barrier instability, and impaired wound healing [36,37,38,39]. In our study, the test product enhanced both superficial and deep hydration while supporting the skin barrier by upregulating key structural and moisture-related proteins. These molecular changes are associated with reduced TEWL and water vapor density, improved skin texture, and faster recovery following physical irritation.

However, the single-arm, open-label design and homogeneous cohort of healthy Korean women limit the generalizability of these findings. Without a placebo or vehicle control, observed effects cannot be definitively attributed to AMP2Na. The positive subjective responses of participants may be influenced by expectation bias. Although several parameters were statistically significant, the modest effect sizes may limit real-world perceptibility; however, consistent improvements across endpoints may collectively enhance overall skin appearance. Furthermore, early reductions in TEWL and erythema should be interpreted cautiously, as they may reflect transient physical effects like occlusion rather than immediate biological repair. Consequently, these short-term findings should be considered preliminary and descriptive. Future blinded, randomized, vehicle-controlled studies in more diverse populations, incorporating additional objective measures (e.g., skin thickness and elasticity) and extended follow-up, are warranted to better reflect biologically meaningful effects and assess potential delayed adverse effects.

Laboratory investigations were similarly limited. The efficacy-focused, translational design did not include direct measurements of intracellular ATP or mitochondrial function, leaving mechanistic links unverified. Additionally, the lack of a vehicle control—including the use of PBS as a baseline comparator in the ex vivo model—makes it impossible to exclude the contributions of other ingredients. Future studies using well-controlled systems with direct metabolic and mitochondrial assays are needed to clarify the specific role and mechanism of AMP2Na.

## 4. Materials and Methods

### 4.1. Test Product

The test product investigated was an AMP2Na-containing product formulated as a milky-white liquid (InnerSignal Rejuvenate Extract, Otsuka Pharmaceutical Co., Ltd., Tokyo, Japan). Table S1 outlines its components.

### 4.2. In Vitro Fibroblast Model

#### 4.2.1. Cell Culture

HDFs, obtained from Thermo Fisher Scientific (Waltham, MA, USA), were cultured in Dulbecco’s Modified Eagle Medium (DMEM; Lonza, Basel, Switzerland) supplemented with 10% Fetal Bovine Serum (Gibco, Waltham, MA, USA) and 1% Penicillin–Streptomycin (Gibco). Cultures were maintained in a humidified incubator at 37 °C with an atmosphere of 5% CO_2_. Cells at passage 6 were used for the experiments.

#### 4.2.2. Cell Viability Assay

HDFs were seeded in 96-well plates (5 × 10^4^ cells/well). Upon reaching >80% confluency, the cells were treated with various concentrations of the test product (0.001%, 0.01%, 0.1%, and 1%, diluted in medium) for 24 h. Cell viability was assessed using CCK-8 assay (Dojindo, Rockville, MD, USA); after 2 h incubation with WST substrate solution at 37 °C, absorbance at 450 nm was measured by a microplate reader (VARIOSKAN LUX; Thermo Fisher Scientific). Cell viability was calculated based on the optical density (OD) relative to the untreated control.

#### 4.2.3. SA-β-gal Assay

To evaluate anti-aging efficacy, replicative senescence in HDFs was induced through 35 serial passages, with cells at passage 6 used as the negative control. HDFs were seeded in 24-well plates (5 × 10^4^ cells/well), grown to >80% confluency, then treated with the test product at different concentrations (0.01%, 0.1%, 1%) for 24 h. The culture medium was removed, and the cells were washed with phosphate-buffered saline (PBS), fixed, and subsequently stained with X-gal solution for 24 h at 37 °C. Blue-stained (SA-β-gal-positive) cells were imaged at 200× (BX43F; Olympus, Tokyo, Japan), and their percentage relative to total cells was calculated, with higher values indicating greater senescence.

#### 4.2.4. Antioxidant Enzyme Activity Assays

HDFs were seeded in 6-well plates at a density of 5 × 10^4^ cells/well and cultured until reaching >80% confluency. To induce oxidative stress, cells were co-treated with 75 µM H_2_O_2_ and the test product at different concentrations (0.01%, 0.1%, and 1%) for 24 h. To assess antioxidant potential, SOD and CAT activities were measured. Culture medium was collected and centrifuged at 2000× *g* for 10 min, and the supernatant was analyzed using commercial SOD and CAT assay kits (BIOMAX, Derwood, MD, USA). Absorbance was measured with a VARIOSKAN LUX reader, and enzyme activities were calculated based on the instructions.

### 4.3. Ex Vivo Human Skin UVB-Induced Aging Model

#### 4.3.1. Human Skin Explant Culture and Treatment Protocol

Human skin tissue was ethically obtained from a healthy donor undergoing elective surgery at Severance Hospital, Seoul, Republic of Korea. The tissue was defatted, washed with PBS, and cut into 1 cm × 1 cm sections, which were divided into three groups: (i) PBS only, (ii) UVB + PBS, and (iii) UVB + test product. UVB irradiation was applied to tissue samples using a UV cross-linker (BLX 312; Vilber Lourmat, Marne-la-Vallée, France) emitting at 312 nm. Irradiation was administered at 100 mJ/cm^2^/day for melanogenesis induction [40] and 300 mJ/cm^2^/day for photoaging induction [41]. The irradiation conditions were controlled by adjusting the exposure time according to the instrument settings to ensure consistent UVB delivery across samples. Immediately after irradiation, either the test product (1%) or PBS was applied topically to the epidermis at a dose of 20 µL per cm^2^ of skin. Following absorption, tissues were cultured in a semi-solid culture medium at 37 °C and 5% CO_2_. UVB irradiation, topical agent application, tissue incubation, and culture medium replacement were performed at 24 h intervals over a total period of 72 h (three cycles). The semi-solid matrix was prepared as described in our previous studies [42,43].

#### 4.3.2. Histological and Histochemical Analyses

After 24 h (for safety assessment) and 72 h (for efficacy assessment) of culture, tissue explants were fixed in 10% formalin, embedded in paraffin, and sectioned for subsequent staining procedures:

- Safety assessment (H&E): Deparaffinized sections were stained with hematoxylin (S3309; Dako, Glostrup, Denmark) for nuclei and eosin (318906; Sigma-Aldrich, St. Louis, MO, USA) for cytoplasm, followed by washing, dehydration, and mounting. The epidermis and papillary dermis were imaged at 400× using an optical microscope (BX43F; Olympus).
- Anti-wrinkle efficacy (MT): Sections were mordanted with Bouin’s solution (BBC Biochemical, Mount Vernon, WA, USA), stained with hematoxylin (H3136; Sigma-Aldrich) for nuclei, biebrich scarlet acid fuchsin (B6008; Sigma-Aldrich) for cytoplasm/muscle, differentiated with phosphomolybdic–phosphotungstic acid (221856, 79690; Sigma-Aldrich), and counterstained with aniline blue to visualize collagen. Images (400×) were analyzed with ZEN software (version 3.4, Carl Zeiss AG, Oberkochen, Germany) to calculate collagen fiber density (%).
- Newly collagen synthesis (HV): Deparaffinized sections were stained with Herovici’s Stain Kit (Scytek Laboratories, Logan, UT, USA) following the manufacturer’s protocol to distinguish young (blue) and mature (red) collagen. After dehydration and mounting, sections were imaged at 400× (BX43F), and neocollagen (blue) within the papillary dermis was quantified using ImageJ (version 1.54g, National Institutes of Health, Bethesda, MD, USA), expressed as the percentage of collagen area relative to total tissue area.
- Elastic fiber density (VVG): Deparaffinized sections were stained with Verhoeff solution (elastic fibers: black), differentiated with 2% ferric chloride (451649; Sigma-Aldrich), treated with 5% sodium thiosulfate, and counterstained with Van Gieson’s solution. Sections were dehydrated, mounted, imaged at 400× (BX43F), and elastic fiber density (%) was quantified using Zen software.
- Skin-whitening efficacy (FM): Deparaffinized sections were stained with the Fontana–Masson Kit (ab150669; Abcam, Cambridge, UK), dehydrated, mounted, and imaged at 400×. Melanin content (black) was quantified with ImageJ as % melanin-positive area relative to total epidermal and dermal area.

#### 4.3.3. Gene Expression Analysis by qRT-PCR

After completing 72 h of AMP2Na treatment, total RNA was extracted from skin tissue samples using TRIzol Reagent (Invitrogen, Carlsbad, CA, USA) after homogenization with TissueLyser II (Qiagen, Hilden, Germany). cDNA was synthesized using the RNA to cDNA EcoDry™ Premix (Clontech, Mountain View, CA, USA). qRT-PCR was then performed using TaqMan Gene Expression Master Mix (Applied Biosystems, Waltham, MA, USA) with TaqMan primers (*filaggrin:* Hs00856927_g1, *loricrin:* Hs01894962_s1, *involucrin:* Hs00846307_s1, *transglutaminase 1 (TGM1)*: Hs00165929_m1; Applied Biosystems). *GAPDH* (Hs02786624_g1; Applied Biosystems) served as the housekeeping gene. Ct values were obtained in real time, and relative mRNA expression was calculated from these values.

#### 4.3.4. IF for Protein Expression Analysis

Following completion of AMP2Na treatment, tissue explants were embedded in Optimal Cutting Temperature compound, snap-frozen and sectioned at 6 µm using a cryostat microtome (Leica Biosystems, Nussloch, Germany). Sections were mounted on silane-coated slides, fixed, permeabilized, blocked, then incubated overnight at 4 °C with primary antibodies against Ki67 (ab15580; Abcam), collagen IV (ab6586; Abcam), laminin (L8271; Sigma-Aldrich), and filaggrin (ab81468; Abcam). After washing, the sections were incubated with secondary antibodies (Goat pAb to Rb IgG (H + L): a11012; Invitrogen, Goat pAb to Rb IgG-FITC: ab6717; Abcam, Goat pAb to Mouse IgG (H + L): a32727; Invitrogen). Nuclei were counterstained with DAPI (Vector Laboratories, Newark, CA, USA) using VECTASHIELD^®^. Fluorescence images (200×) were acquired using a Zeiss LSM 700 confocal microscope (Oberkochen, Germany) and analyzed with Zen software. Ki67 was quantified as the percentage of Ki67-positive nuclei among total basal keratinocytes, whereas collagen IV and laminin were assessed by mean fluorescence intensity within the basement membrane zone, and filaggrin within the epidermal barrier.

#### 4.3.5. ELISA

HA production was assessed after 72 h of treatment by homogenizing explants with TissueLyser II, centrifuging (2000× *g*, 10 min), and collecting the supernatant. Total protein was measured with a BCA Protein Assay Kit (Sigma-Aldrich). HA was quantified using a Hyaluronan Quantikine ELISA Kit (R&D Systems, Minneapolis, MN, USA) with absorbance read on the VARIOSKAN LUX reader and concentrations calculated from a standard curve, corrected for dilution, and normalized to total protein.

All experiments were conducted at least three times.

### 4.4. Clinical Trial 1: Comprehensive Evaluation of Anti-Aging and Skin Tone Efficacy

#### 4.4.1. Study Design and Participants

A 12-week, exploratory, clinical trial was conducted at the Global Medical Research Center in Seoul, Republic of Korea. A total of 23 female participants aged 35–59 years with facial hyperpigmentation were enrolled, and the sample size was validated according to the Ministry of Food and Drug Safety guidelines. Eligible participants were free of acute or chronic illness, including skin disease, and able to complete the trial. Exclusion criteria included pregnancy or lactation; active lesions, infections, or allergies at test sites; hypersensitivity to cosmetics or medications; systemic steroids or phototherapy within one month; and cosmetic/dermatologic procedures (peels, botox, fillers, lasers) or use of similar products on test sites within three months.

#### 4.4.2. Study Procedures

Participants applied the test product (3–4 pumps) to the entire face twice daily after cleansing for 12 weeks. Efficacy and safety were assessed at baseline and multiple follow-up visits. Before all instrumental measurements, participants cleansed their face and acclimatized for 30 min in a controlled environment (20–24 °C; 45–55% relative humidity).

#### 4.4.3. Efficacy Evaluations

Objective measurements:

The following devices were used to assess changes in skin condition BT, IAT (within 10 min after application), at W1, W4, W8, and W12 after treatment:

Wrinkles and texture were assessed using an Antera 3D CS camera (Miravex, Dublin, Ireland). Wrinkle depth (mm) was measured in the “star zone” (crow’s feet, nasolabial folds, glabella) and on the neck, while skin texture (Ra, arbitrary unit (AU)) was quantified on the left cheek. A predefined region of interest (ROI) was set on the captured images for analysis. The same ROI was automatically aligned and applied across different time points using the device software to ensure that the identical area was analyzed. For quantitative assessments, each parameter was measured three times, and the average value was used for analysis. To minimize variability, repeated measurements were controlled so that deviations did not exceed predefined acceptance criteria.

The next set of equipment was used to evaluate changes BT, 3 days, W1, W4, W8, and W12 after treatment:

- Skin tone (standard light), radiance (parallel-polarized light), and transparency (cross-polarized light) were assessed using VISIA-CR imaging (Canfield Science, Ltd., Parsippany, NJ, USA) combined with IMAXPlus software version 11.0.

We also used the following set of devices to quantify the progress of other skin qualities BT, W1, W4, W8, and W12 after treatment:

- Superficial pigment area was assessed using an Antera 3D CS camera (Miravex, Dublin, Ireland) (mm^2^).
- Blemishes within the stratum corneum were evaluated using a D-Squame standard sampling disk applied with uniform pressure via the D-Squame Pressure Instrument (Clinical and Derm LLC., Dallas, TX, USA). The sampled area was analyzed with Antera 3D CS. A reduction in the measured area (mm^2^) under Melanin Mode indicated improvement in blemishes.
- Deep pigmentation (UV mode) was evaluated using VISIA-CR imaging.
- Superficial skin hydration was measured on the cheek using a Corneometer CM825 (Courage + Khazaka, Köln, Germany) (A.U.); higher values indicate greater hydration.
- Deep skin hydration was measured with Moisturemeter D Compact (Delfin, Kuopio, Finland) (PWC%); higher values indicate greater hydration.
- TEWL was measured on the cheek using a Tewameter TM300 Hex (Courage + Khazaka, Germany). Each measurement lasted 25 s, and the average of the final 3 s was used for evaluation to assess skin barrier integrity (g/m^2^h), with lower values indicating improved skin barrier function. The same device was used to evaluate the skin’s water-holding capacity (g/m^3^).
- Skin exfoliation was assessed by collecting corneocytes with D-squame disks (Clinical and Derm LLC.) and imaging with Visioscan VC98 (Courage + Khazaka, Germany) to calculate the Desquamation Index (%), with lower values indicating improved corneocyte content.
- Midface lifting was assessed using the F-ray system (BEYOUNG Co., Ltd., Seoul, Republic of Korea). Lateral facial images (60°) were analyzed using Moiré fringes, with the horizontal-to-vertical ratio of the second contour line; lower ratios indicated improved midface lifting.

Subjective measurements: Product efficacy was evaluated through 8 items, using a 5-point Likert scale survey (5 = strongly agree, 4 = agree, 3 = neutral, 2 = disagree, 1 = strongly disagree) completed by all participants at weeks 1, 4, 8 and 12. Responses with a score of 4 and 5 were considered positive and were reported accordingly.

The safety of the test product was assessed by recording and analyzing all adverse events (e.g., itching, stinging, or rash) reported or observed during the study period, with incidence rates calculated accordingly.

Compliance was tracked via participant diaries, and data from participants with ≥ 80% compliance were included in the analysis.

### 4.5. Clinical Trial 2: Evaluation of Skin Turnover and Soothing Efficacy

#### 4.5.1. Study Design and Participants

The second trial was an exploratory, prospective, single-center study of 22 female participants, conducted at the Global Medical Research Center. Inclusion/exclusion criteria, acclimation period, procedures for efficacy, safety, and adverse event assessments were identical to the first trial.

#### 4.5.2. Study Procedures

The study consisted of two primary evaluations conducted on the forearms of each participant:

- Skin soothing was conducted on the left forearm over one week using two adjacent 2 × 2 cm sites (test product-treated vs. non-treated (control)). Baseline TEWL and redness were measured, and both sites were barrier-disrupted with 10–15 tape strips to induce comparable irritation, and the product was applied to the designated site. TEWL (Tewameter TM300) and redness (Antera 3D, decrease in hemoglobin concentration [A.U.] indicating reduced erythema) were reassessed immediately, at 3 days, and at 1 week.
- Skin turnover was conducted on the right forearm over two weeks using a 2 × 2 cm test site and adjacent control. After baseline fluorescence, the stratum corneum was stained with a 15 µL dansyl chloride patch 5%. After 24 h, initial fluorescence was recorded, after which participants applied the product twice daily for two weeks. Fluorescence intensity was measured at 3 days, W1, and W2 using the Mark-Vu imaging system (PSIPLUS Co., Ltd., Suwon, Republic of Korea) in UV mode, with a faster decline indicating an accelerated turnover rate.

Subjective measurements: Product efficacy was evaluated using a single item on a 5-point Likert scale, as described in Clinical trial 1.

### 4.6. Statistical Analyses

All analyses were performed using IBM SPSS Statistics, version 25.0, with statistical significance set at *p* < 0.05. Graphs were created using GraphPad Prism version 10.2.3. Data are presented as mean ± standard deviation. For clinical trials, after testing for normality, within-group comparisons between two time points (e.g., before damage vs. after damage, before staining vs. after staining) were performed using a paired *t*-test for normally distributed data or a Wilcoxon signed-rank test otherwise. Comparisons across three or more time points (e.g., weeks 1, 4, 8, and 12 vs. before treatment) used repeated measures ANOVA or the Friedman test with Bonferroni correction, depending on normality. For in vitro and ex vivo studies, normality was first assessed, followed by an independent samples t-test to compare two experimental groups.

## 5. Conclusions

In conclusion, this study suggests that the AMP2Na-containing product was well tolerated and associated with improvements in multiple clinical and biological parameters related to skin aging. By potentially supporting epidermal metabolic activity, it might accelerate turnover, promote natural melanin clearance, and reinforce the skin barrier. These actions collectively improve tone, firmness, and hydration while reducing photodamage. The formulation thus offers a physiology-aligned, well-tolerated approach to skin rejuvenation. Further large-scale, vehicle-controlled clinical studies are warranted to confirm these findings and extend their applicability across diverse populations.

## Figures and Tables

**Table 1:** Questionnaire results on skin rejuvenation after the test product use.

Item No.	Question	Time Point	Points (n)					Positive Response Rate (≥4 Points)
			1	2	3	4	5	
1	Do you feel that wrinkles (around the eyes, forehead, nasolabial folds, neck) have improved?	1 week	0	0	4	19	0	82.61%
		4 weeks	0	0	1	20	2	95.65%
		8 weeks	0	0	0	19	4	100%
		12 weeks	0	0	0	20	3	100%
2	Do you feel that neck wrinkles have improved?	1 week	0	0	3	20	0	86.96%
		4 weeks	0	0	4	17	2	82.61%
		8 weeks	0	0	2	18	3	91.3%
		12 weeks	0	0	3	18	2	86.96%
3	Do you feel that your skin has become softer and more hydrated?	1 week	0	0	1	18	4	95.65%
		4 weeks	0	0	0	13	10	100%
		8 weeks	0	0	0	13	10	100%
		12 weeks	0	0	0	12	11	100%
4	Do you feel that skin texture has improved?	1 week	0	0	1	21	1	95.65%
		4 weeks	0	0	0	19	4	100%
		8 weeks	0	0	0	14	9	100%
		12 weeks	0	0	0	13	10	100%
5	Do you feel that your skin looks brighter or more radiant?	1 week	0	0	2	17	4	91.3%
		4 weeks	0	0	3	14	6	86.96%
		8 weeks	0	0	1	15	7	95.65%
		12 weeks	0	0	1	15	7	95.65%
6	Do you feel that the spots on your skin have lightened?	1 week	0	0	8	15	0	65.22%
		4 weeks	0	0	4	17	2	82.61%
		8 weeks	0	0	3	18	2	86.96%
		12 weeks	0	0	4	16	3	82.61%
7	Do you feel that the mid-face has been lifted?	1 week	0	0	2	20	1	91.3%
		4 weeks	0	0	3	15	5	86.96%
		8 weeks	0	0	0	20	3	100%
		12 weeks	0	0	2	18	3	91.3%
8	Do you feel that dead skin cells are less noticeable and your skin looks fresher?	1 week	0	0	2	19	2	91.3%
		4 weeks	0	0	1	19	3	95.65%
		8 weeks	0	0	1	16	6	95.65%
		12 weeks	0	0	2	16	5	91.3%

1 = strongly disagree, 2 = disagree, 3 = neutral, 4 = agree, 5 = strongly agree.

**Table 2:** Questionnaire results on skin recovery after the test product use.

Question	Time Point	Points (n)					Positive Response Rate (≥4 Points)
		1	2	3	4	5	
Do you feel that skin irritation caused by external stimuli has been relieved?	Immediately after treatment	0	0	5	13	4	77.27%
	After 3 days	0	0	3	16	3	86.36%
	After 1 week	0	0	1	16	5	95.45%

1 = strongly disagree, 2 = disagree, 3 = neutral, 4 = agree, 5 = strongly agree.

## Data Availability

The original contributions presented in this study are included in the article. Further inquiries can be directed to the corresponding author.

## Supplementary Materials

The following supporting information can be downloaded at: https://www.mdpi.com/article/10.3390/ijms27114840/s1.

## Abbreviations

The following abbreviations are used in this manuscript:

AMP2Na	Disodium Adenosine Monophosphate
AMP	Adenosine Monophosphate
ATP	Adenosine Triphosphate
AU	Arbitrary unit
cAMP	Cyclic Adenosine Monophosphate
HDFs	Human Dermal Fibroblasts
ECM	Extracellular Matrix
UV/UVB	Ultraviolet/Ultraviolet B
ROS	Reactive Oxygen Species
DMEM	Dulbecco’s Modified Eagle Medium
PBS	Phosphate-Buffered Saline
CCK-8	Cell Counting Kit-8
WST	Water-Soluble Tetrazolium Salt
OD	Optical Density
SA-β-gal	Senescence-Associated β-Galactosidase
H_2_O_2_	Hydrogen peroxide
SOD	Superoxide Dismutase
CAT	Catalase
H&E	Hematoxylin and Eosin
MT	Masson’s Trichrome
HV	Herovici Stain
VVG	Verhoeff–Van Gieson
FM	Fontana–Masson
IF	Immunofluorescence
qRT-PCR	Quantitative Real-Time Polymerase Chain Reaction
mRNA	Messenger RNA
cDNA	Complementary DNA
GAPDH	Glyceraldehyde-3-Phosphate Dehydrogenase
ELISA	Enzyme-Linked Immunosorbent Assay
HA	Hyaluronic Acid
BCA	Bicinchoninic Acid Assay
W	Week/Weeks
TEWL	Transepidermal Water Loss
DEJ	Dermal–Epidermal Junction
TGM1	Transglutaminase 1
DAPI	4′,6-Diamidino-2-Phenylindole
IRB	Institutional Review Board
SPSS	Statistical Package for the Social Sciences
ANOVA	Analysis of Variance
PWC	Percentage Water Content
