# Supplementary material for: Translational Evaluation of a Disodium Adenosine Monophosphate (AMP2Na)-Based Topical Formulation for Physiology-Aligned Skin Rejuvenation: Integrated In Vitro, Ex Vivo, and Clinical Evidence
Source: Int J Mol Sci. 2026 May 27;27(11):4840. doi: 10.3390/ijms27114840 (PMC13256818; doi:10.3390/ijms27114840)
Supplement: Supplementary file 1 [file ijms-27-04840-s001.zip › Supplementary table.pdf]

Table S1. Ingredients in AMP2Na

Ingredient Name
Disodium adenosine phosphate
Dipotassium glycyrrhizate
Glycerin
Squalane
Alcohol
Polyglyceryl-10 isostearate
Acrylates/C10-30 alkyl acrylate crosspolymer
Sodium isostearoyl lactate
Polyglyceryl-10 stearate
Methylparaben
Sodium hydroxide
Butylparaben
Water
